# 3D μEFPocket for Multimodal Communications with Brain Organoids

**DOI:** 10.21203/rs.3.rs-9510258/v1

**Published:** 2026-08-31

**Authors:** Yihang Wang, Miguel Cuevas, Zhihan Zhang, Gooyoon Chung, Jingze Zhang, Yilan Yin, Qianzhi Luo, Will Lipman, Josh Henderson, Lucas Lu, Weixiang Zeng, Anran Zhang, Changxin Jia, Haiwen Luan, Jason Stein, Wubin Bai

**Affiliations:** UNC at Chapel Hill; UNC at Chapel Hill; UCSD; UNC at Chapel Hill; UNC at Chapel Hill; UNC at Chapel Hill; UNC at Chapel Hill; UNC at Chapel Hill; UNC at Chapel Hill; UNC at Chapel Hill; UNC at Chapel Hill; University of North Carolina at Chapel Hill; UNC at Chapel Hill; Northwestern University; UNC-Chapel Hill; The University of North Carolina at Chapel Hill

## Abstract

Human brain organoids are powerful three-dimensional models for studying neural development, disease mechanisms, and pharmacological responses, but precise spatiotemporal chemical modulation and activity recording within intact organoids and in a simultaneous fashion is difficult due to their three-dimensional (3D) complexities. Here, we introduce the microElectroFluidicPocket (μEFPocket), a conformal, ultrathin microfluidic 3D biointerface that integrates microscale spatiotemporal control of chemical and electrical stimulation in human cortical organoids with simultaneous optical and electrical readout. Fabricated entirely from parylene C using standard wafer-scale deposition, photolithography, and etching, μEFPockets form integrated, mechanically compliant architectures that wrap around spherical organoids without attachment factors, enabling repeated use of the same organoid. Engineered and independently addressable microchannels enable localized, time-locked delivery of neurotransmitters with hundreds-micron spatial resolution and second-level temporal control while preserving global culture conditions. Using glutamate delivery or electrical stimulation of GCaMP-expressing human cortical organoids, we demonstrate spatially localized calcium responses at regional and single-neuron resolution, revealing network propagation and stimulus-dependent adaptation. This scalable platform provides a foundation for spatially patterned pharmacology, multimodal biointerfaces, and closed-loop functional assays in organoids.

## Introduction

Human brain organoids have transformed neuroscience by enabling experimental access to human-derived brain cells, which recapitulate key aspects of neural development, cellular diversity, and emergent network activity^1–3^. These systems offer unprecedented opportunities to investigate neurodevelopmental disorders, disease mechanisms, and therapeutic responses in a controlled and scalable experimental setting^4,5^. In parallel, substantial efforts have been devoted to integrating engineered biointerfaces with organoid cultures to enable long-term electrophysiological monitoring and functional perturbation. Two-dimensional microelectrode arrays (MEAs), including complementary metal-oxide-semiconductor (CMOS)-based platforms^6–8^, have been widely adopted to record spontaneous and evoked electrophysiological activity, enabling both population-level and single-unit analyses as well as pharmacological screening. Microfluidic platforms, conventionally in a planar form, have further enhanced organoid culture by supporting chemical gradient formation, and increasing throughput for drug testing^9–12^. Despite these advances, most commercially available platforms to interface with organoids remain fundamentally planar and rigid, leading to a pronounced mechanical and geometrical mismatch with soft, spherical organoids^13–15^. As a result, electrical recording and stimulation are typically confined to limited planar regions in contact, while missing critical insights from those curvilinear regions of organoids in three dimensions (3D). Furthermore, conventional chemical interactions are deployed across the entire organoid. This lack of spatial precision represents a critical limitation, as brain development and function are inherently governed by spatially organized chemical gradients and region-specific signaling. Studies have shown that localized morphogen delivery can drive region-specific patterning and functional heterogeneity in organoids, underscoring the importance of spatially controlled biochemical environments^16–21^. In contrast, bulk stimulation obscures these relationships by coupling all regions to the same chemical input, preventing causal mapping between local perturbations and neural responses. Therefore, overcoming these limitations requires biointerfaces capable of precise, localized, and temporally controlled delivery of chemicals while maintaining mechanical compliance and conformal interfacing with 3D tissues.

In recent decades, flexible and mesh-like bioelectronic devices have substantially expanded the capabilities of electrophysiological recording and stimulation in organoid systems^22–24^. Conformal surface-contact MEAs, fabricated on ultrathin polymer substrates, have enabled improved mechanical matching with soft organoids, allowing stable long-term recordings, reduced tissue damage, and enhanced signal fidelity compared to rigid planar arrays^25–32^. These platforms support surface-resolved mapping of spontaneous and evoked activity and have been instrumental in characterizing network maturation and pharmacological responses. In parallel, mesh-like bioelectronic architectures have been developed to grow within organoids during early differentiation, enabling integration of electrodes with the tissue and providing access to deep network dynamics that are otherwise inaccessible from the surface^33–40^. While some flexible and mesh-based MEA systems have incorporated fluidic components to introduce chemical stimuli, drug delivery in these platforms is typically mediated through bulk media exchange or global perfusion, resulting in limited spatial and temporal precision^41^. Consequently, chemical modulation remains largely decoupled from the fine-grained electrical access provided by these devices, restricting the ability to perform localized, time-resolved pharmacological perturbations within intact 3D organoids.

Existing microfluidic platforms, though widely used to construct artificial organs and organ-on-a-chip systems, are predominantly in planar architecture, making them not well suited for comprehensive interface with 3D spherical organoids^9,42,43^. In such cases, organs and organoids are typically permanently cultured within the device^42,43^ or mechanically immobilized using hydrogels^20^ or suturing approaches^16^, which constrain their natural morphology and limit experimental flexibility. Moreover, seamlessly integrating microfluidics with flexible or mesh-like MEA platforms in a 3D bio-inspired architecture remains a challenge because traditional approaches typically require complex molding, assembly, and bonding processes that limit fabrication scalability, compromise mechanical compliance and conformal electrode–tissue contact, and limit quantitative and spatial precision in chemical delivery^44–47^.

Progress in functional interrogation of organoids has been constrained by the lack of interfaces capable of delivering and collecting biochemical cues with precise spatial and temporal control over a long term while preserving tissue integrity. Moreover, platforms that combine localized chemical perturbation with simultaneous optical readout of neural activity remain absent, preventing direct correlation between spatiotemporal drug exposure and functional response within intact organoids. The integration of electronics, fluidics, and optical components in a flexible tissue-like device is required for next-generation organoid interfacing. Here, we introduce a series of 3D, flexible microfluidic and microelectrofluidic devices termed μFPockets and μEFPockets, respectively, that enable conformal electrical and biochemical modulation of human cortical organoids (hCOs) with real-time optical imaging. The platform is fabricated entirely from ultrathin Parylene C (PPX-C, an FDA-approved Class VI biocompatible, biostable polymer), forming a multilayer architecture that integrates flexible backbones, embedded microchannels, and spatially distributed fluidic inlets and outlets. Importantly, all structural and fluidic features are realized using standard deposition–photolithography–etching processes, enabling complex channel patterning and precise inlet–outlet control without specialized equipment or nonstandard fabrication steps. This architecture allows the device to wrap around spherical organoids without inducing significant deformation while enabling on-demand, region-specific delivery of neurotransmitters or drugs with second-level temporal precision. This capability enables seamless co-fabrication of microfluidic and microelectronic interfaces within a single device. Furthermore, the optical transparency and low fluorescence of PPX-C, together with the customized design of the media reservoir, permit seamless compatibility with in situ calcium (Ca) imaging, enabling simultaneous stimulation and functional readout across multiple regions of the same organoid. Moreover, conformal interfacing and chemical delivery using the μEFPocket do not require any attachment factors or surface immobilization, allowing the organoid to be easily inserted and removed multiple times without compromising viability or functionality. Based on this feature, we demonstrated the performance of μEFPockets through a series of Ca imaging experiments of real-time chemical delivery to GCaMP8s expressing hCOs, demonstrating precise spatiotemporal control of drug release. Beyond localized stimulation, this platform enables high-resolution spatial control of chemical delivery when interfaced with hCOs in liquid media through engineered microfluidic geometries and independently addressable inlet-outlet channels. By adjusting outlet placement, flow rates, and delivery modes, local concentration levels and gradients can be precisely regulated locally at the tissue interface, providing a foundation for both spatiotemporal control of drug distribution and chemical patterning within organoids. Such control creates opportunities to probe neuromodulator gradients, synaptic plasticity, and network adaptation under dynamic stimulation paradigms, while also enabling studies of developmental signaling, region-specific toxicity, and heterogeneous therapeutic responses in organoids^17,48–50^. By integrating 3Dl mechanical compliance, localized microfluidic control, and real-time optical readout within a scalable system, this work introduces a new class of conformal, multimodal organoid interfaces and lays the foundation for patterned pharmacological interrogation and closed-loop chemical–functional assays in human brain organoids, with broad relevance to neuroengineering and translational neuroscience.

## Results and Discussion

Fig. 1 demonstrates the concept, architecture, and functional capabilities of the μFPocket platform for localized chemical stimulation of 3D neural organoids with simultaneous optical readout. Conventional pipette-based drug delivery (Fig. 1a, left) introduces substantial spatial and temporal uncertainty due to diffusion, fluid convection, and mechanical disturbance of the tissue, which limits reproducibility and precludes precise correlation between stimulus timing, spatial location, and cellular response. In contrast, the μFPocket enables confined, time-locked, and minimally invasive delivery of chemical stimuli directly to the organoid surface while preserving multimodal access for either electrical stimulation or optical imaging (Fig. 1a, right). This integrated approach addresses a key technical limitation in studying localized pharmacological modulation in 3D tissue models.

To systematically explore the design space enabled by our fabrication technique, we developed a family of microfluidic and microElectroFluidicPocket (named as μFPocket-1 to 5, μEFPocket-1 to 3, respectively) with varying geometries, channel architectures, and interfacing strategies, as summarized in **Table S1**. These variants are derived from a common planar microfabrication framework but are differentiated through mechanical programming and fluidic layout to achieve distinct functionalities, including conformal wrapping, spatially confined delivery, adaptive contact, and multimodal integration with flexible microelectrode arrays (fMEAs). This design diversity highlights the versatility and scalability, demonstrating that a single fabrication paradigm can be extended to address a wide range of experimental needs, from localized chemical stimulation to combined chemical–electrical interrogation of 3D tissues.

Structurally, μFPocket-1 is realized as a planar, lithographically defined PPX-C microfluidic network (2D structure shown in **Fig. S1**) that is subsequently assembled into a 3D organoid-interfacing structure (Fig. 1b–c). Its 2D structure features a mesh-like fluidic network with 120-μm line width and 20-μm channel width, up to eight microfluidic channels. The outlets of channels are densely distributed in the center within an area of ~1.75 mm^2^, including up to 8 separate microfluidic channels (**Fig. S2**). **Supplementary Movie 2** shows functionality of the microfluidic array of μFPocket-1. μFPocket-1 is assembled through a hybrid 2D-to-3D transformation that combines lithographically defined PPX-C microfluidics with modular reservoir integration (**Fig. S3**). Following wafer-scale fabrication and release (**Fig. S7–8**), the ultrathin PPX-C microfluidic sheet is mechanically compliant and can be deformed without loss of channel integrity. Drug and media reservoirs are created using PDMS structures bonded to glass substrates, which interface with the microfluidic inlets through precisely aligned channel connectors, enabling robust fluidic coupling while preserving optical transparency. Notably, optical inspection and dye perfusion tests confirm that PPX-C microchannels remain fully sealed after PDMS encapsulation (**Fig. S4**). Even under extreme folding, the PPX-C microfluidics show no observable leakages. During assembly, the planar microfluidic network is guided into a 3D configuration by controlled compressive buckling of the serpentine mesh segments, which act as compliant hinges and strain-relief elements. This transformation creates a central pocket that protrudes out of plane and can enclose organoids with a diameter ranging from 1–3.5 mm, while the peripheral regions remain mechanically anchored to the substrate through bonding sites (Fig. 1c, **Fig. S5**). In air (Fig. 1d–e, **Fig. S5a**) and in water (**Fig. S5b**, **Supplementary Movie 3**) experiments confirm that the assembled 3D structure maintains sealed channels, reliable fluidic delivery, and stable shape. Time-lapse imaging reveals sequential and independently addressable release from eight individual microfluidic channels, with dye delivery progressing in a programmed manner across stages i–viii (**Fig. S6**). By adjusting folding positions and compression distance, a range of opening angles of the pocket can be achieved (**Fig. S5c-d**). Finite element analyses (FEAs) further demonstrate that mechanical deformation during the assembling process is uniformly distributed across the serpentine network without localized stress concentration (maximum principal strain below 1%), thereby preserving channel patency and structural integrity. In addition, the peripherally separated fluidic channel adapters enhance accessibility to organoids during loading and unloading procedures, while maintaining optical transparency and enabling long-term operational stability.

When an organoid is positioned within the μFPocket (Fig. 1f), the device stably hosts the organoid at its center and establishes controlled contact between the microfluidic outlets and the organoid surface through a flexible, mesh-like interface without visible deformation or displacement. The absence of gross tissue compression or leakage indicates that the system provides gentle yet reliable mechanical and fluidic interfacing, which is critical for long-term viability and functional measurements in delicate neural constructs. Importantly, the open mesh architecture enables unhindered transport of nutrients, oxygen, and waste between the organoid and the surrounding medium, preventing a potential contact-limited diffusion micro-environments. In addition, the sparse, lattice-like geometry maximizes optical clearance by minimizing material coverage above the tissue, allowing high-quality fluorescence imaging and, when electrodes are embedded within the mesh, reducing optical obstruction and shadowing artifacts. Meanwhile, the intrinsic biocompatibility and chemical inertness of PPX-C ensure the safety and stability of long-term interfacing. Functionally, Fig. 1f demonstrates the capability of the μFPocket to evoke localized and temporally resolved neural activity within a living organoid, validating that this mechanically compliant, optically transparent, and transport-permissive interface supports precise chemical stimulation without compromising tissue health or imaging fidelity. Additionally, Fig. 1e, g highlights a conformal μFPocket design incorporating multiple independently addressable microfluidic channels distributed around the organoid surface, illustrating the scalability and architectural versatility of the platform. This design enables spatial multiplexing of chemical inputs while preserving mechanical compliance and conformal contact. Notably, its configuration incorporates shared outlet channel architectures, enabling coordinated chemical delivery and withdrawal for highly confined and high-precision chemical stimulation. Furthermore, Fig. 1h presents an assembled μEFPocket system that integrates both electrical and fluidic connectors within a single device, demonstrating compatibility with simultaneous electrophysiological recording and localized chemical delivery. In this configuration, the separation of electronic and fluidic interconnects ensures independent optimization of signal integrity and fluidic control, facilitating robust multimodal interrogation. These examples emphasize the diversity and modularity of the μFPocket platform, which will be explored in greater detail in Fig. 2 and Fig. 3.

Fig. 2 demonstrates the lithographically defined, all-on-wafer fabrication techniques of integrated PPX-C-based microfluidics and the mechanical integrity of the example structures under extreme deformation. Unlike conventional soft-lithography or bonded multilayer microfluidics, which are constrained by alignment accuracy, pattern density, and interfacial delamination, the present approach realizes microfluidic networks entirely through wafer-scale microfabrication with standard photolithographic patterning and thin-film deposition. As illustrated in Fig. 2a and **Fig. S7–8**, the microfluidic structures are formed by sequential deposition of a bottom PPX-C layer, lithographically defined sacrificial channel layers, top PPX-C encapsulation, and a patterned hard mask for outline etching. The sacrificial channel layers, consisting of photoresist, are completely removed by post-fabrication dissolution (**Supplementary Movie 1**). This process yields fully integrated, sealed microchannels whose geometry is defined with micrometer-scale precision, enabling deterministic control over channel width, curvature, branching topology, and spatial registration with other functional elements. The optical image in Fig. 2b confirms the high fidelity of the fabricated patterns, including smooth outline boundaries and consistent channel dimensions, highlighting the suitability of this technique for dense, complex, and reproducible microvascular-like network architectures. In addition to microfluidic functionality, the same all-on-wafer fabrication strategy enables the integration of microelectrode arrays (MEAs) within the PPX microfluidic architecture (Fig. 2c–d, **Fig. S9**). By embedding patterned Cr/Au electrodes between successive PPX layers, electrical interfaces are spatially registered to the microfluidic channels with micrometer precision while remaining fully encapsulated and mechanically compliant. This integration eliminates the need for post hoc alignment or adhesive bonding between fluidic and electronic components, thereby improving fabrication yield, device reproducibility, and long-term stability in biofluidic environments.

We first validate the fabrication method with some microfluidic and microelectrofluidic test devices (μFTest-1, μFTest-2, and μEFTest-1), showing exceptional mechanical compliance and resilience (Fig. 2e–h). This is essential for interfacing with soft biological tissues and for deployment in confined or dynamically moving environments. Fig. 2e–f shows that the mesh-like microfluidic device (μFTest-1) can withstand continuous 360° twisting (Fig. 2c) and 180° bending (Fig. 2d) without fracture, delamination, or flow interruption. **Fig. S11** shows the layouts of microvascular fluidic network, showcasing structure adjustability with line widths ranging from 80–650 μm, channel width from 10–300 μm. The standard thickness of each PPX-C layer is 5 μm, which can also be adjusted to increase flexibility or rigidity. The positive templating of microfluidic channel enables precise control of channel thickness via modulating spin-coating parameters, ranging from 7–25 μm. The intertwined serpentine geometry distributes strain across multiple compliant segments, thereby minimizing localized stress concentration. Finite element analyses (Fig. 2g, **Fig. S12**) corroborate the structural integrity with a strain (~0.2%) far below material failure thresholds (yield elongation up to 2.9%)^51,52^, even under extreme torsional and bending deformations. We also validate the mechanical integrity of PPX-C microelectrofluidics by two distinct designs, showing deterministic three-dimensional folding and bending of planar microfluidic layouts into complex 3D structures (Fig. 2g–h, **Fig. S13**)^53^. By encoding curvature, hinge regions, and asymmetric stiffness directly into the design of planar precursor structure, the devices self-organize into well-defined 3D configurations upon release, forming compact 3D meso-structures with integrated microelectrofluidic channels. These structures can be bent, folded, and manipulated, reversibly, without loss of structural integrity or channel patency, demonstrating compatibility with minimally invasive delivery and constrained anatomical environments (**Fig. S14**). The fluidic functions of these designs remain normal even when enclosed in a thin medical catheter (as shown in **Fig. S14**), demonstrating stable chemical delivery (**Supplementary Movie 5**). These examples presented in Fig. 2 validate both the mechanical robustness and architectural versatility of the proposed monolithic microfluidic fabrication approach.

**Fig. S15–16** shows the 2D layouts of μEFPockets 1–3, featuring ultracompact structures with up to 96 MEA channels and three microfluidic channels. The vertical overlay greatly increases component density while preserving mechanical flexibility. The opening regions of microelectronics and microfluidics are separated to avoid interference. Only 2–5 μm of thickness is introduced into the vertical structure, while the device remains stable upon extreme structure deformation, as illustrated in Fig. 2i and **Fig. S17**. The resulting platform supports simultaneous localized chemical delivery and electrophysiological stimulation and recording (**Supplementary Movie 4**, **Fig. S18**) within a single device. Our designs separate the connections of microfluidic and microelectronics, ensuring no interference between different functionalities. Optional electroplating of platinum black (PtB) is also compatible with the fabrication process (Fig. 2j–l, **Fig. S10**). This coating significantly reduces electrochemical impedance and improves signal quality of electrophysiological recording.

Fig. 3 illustrates a family of μFPockets derived from a common, wafer-scale planar microfluidic architecture but differentiated through finite-element-analysis (FEA)-guided mechanical design to achieve distinct conformal interfacing strategies. All μFPocket variants are lithographically defined in two dimensions prior to release (Fig. 3a–c), ensuring high manufacturability and precise control over channel geometry, while their three-dimensional structures are programmed through spatial modulation of outline geometry, bonding sites, and connectivity (**Fig. S19**). μFPocket-2 exemplifies a conformal wrapping strategy rooted in the map projection problem, in which the pocket section is designed by partitioning a sphere into six compliant petals that fold upward through pre-stretching, releasing, and controlled buckling (Fig. 3d). Mechanical bonding at the peripheral region stabilizes the folded structure and maintains continuous contact with the organoid surface (Fig. 3e). Optical imaging and FEA studies confirm that this geometry securely cradles a millimeter-scale organoid phantom while releasing strain across the serpentine arms and avoiding localized compression of the tissue (Fig. 3f–g). This design enables uniform circumferential contact and is well-suited for symmetric chemical interfacing or distributed stimulation. **Fig. S20** and **S21** further elucidate the mechanical adaptability and tunability of μFPocket-2, enabled by FEA-guided structural design. Optical characterization demonstrates that a single μFPocket-2 geometry can stably accommodate spherical organoid phantoms spanning a broad size range (diameter ≈ 1.3, 2.0, and 2.9 mm) while maintaining conformal surface contact in both standby and active delivery states (**Fig. S20a–b**). The broad accommodability is enabled by rationally designed compliant petal structures that undergo elastic deformation, allowing simultaneous adjustment of curvature and contact area to accommodate organoids of varying sizes without inducing excessive local stress. FEA studies further demonstrate that deterministic control over the final three-dimensional configuration can be achieved by systematically tuning the bonding sites and compression distance (**Fig. S20c**). A comprehensive parametric FEA across multiple sphere diameters (**Fig. S21**) identifies a trade-off between conformal contact, strain distribution, and mechanical stability, ensuring that the strain of the functional region remains below material failure thresholds (2.9%) throughout the entire size range. Collectively, these results establish that the mechanics-guided assembly strategy enables a single planar precursor design to robustly interface with heterogeneous organoid geometries, offering a versatile and scalable platform for conformal chemical delivery to diverse 3D tissues. μFPocket-4 (Fig. 3h) follows the same conformal wrapping strategy as μFPocket-2 (Fig. 3f–g), in which deterministic three-dimensional folding is achieved through mechanically programmed planar geometries and peripheral bonding; however, μFPocket-4 differs in its microfluidic channel architecture and routing, which are reconfigured to enable shared channel outlets (Fig. 3i–k). This configuration provides the capability of ultrahigh spatial resolution in controlling concentration gradients of multiple drugs, or for minimizing the affected stimulation area through coordinated drug delivery and withdrawal around the outlet (delivering-removal mode). Finite-element analysis (Fig. 3l) confirms that the buckling behavior of μFPocket-4 is encoded directly in the planar layout through predefined hinge regions and stiffness gradients controlled by cross-section width, yielding predictable three-dimensional configurations while maintaining strain below material failure thresholds.

μFPocket-5, by contrast, adopts an adaptive contacting strategy based on cantilevered microfluidic probes (Fig. 3m), allowing the interface to passively adjust to variations in organoid size, position, and surrounding media condition. This design additionally supports continuous media refreshing through integrated flow paths (Fig. 3n), enabling stable contact in both air–liquid and fully submerged environments (Fig. 3o–p, **Supplementary Movie 6**). The pronounced shape adaptability under externally applied fluid drag (Fig. 3q) highlights the ability of this architecture to maintain functional interfacing under dynamic mechanical conditions. Together, these designs demonstrate that diverse conformal, directional, and adaptive biointerfaces can be deterministically encoded into planar PPX-C microfluidics through mechanical programming, enabling tailored chemical access to complex three-dimensional tissues without sacrificing wafer-scale manufacturability. The outlet geometries of the μFPocket microfluidic channels are rationally engineered to control local flow fields and chemical transport near the tissue interface (**Fig. S22**), confirmed by optical imaging and FEA simulations, including terminal, side, top, and dual-side outlet configurations. In μFPocket-1, side- and top-emitting outlets regulate main flow streams, resulting in preferential diffusion along the tangential direction of the interface, whereas dual-side outlets enable symmetric diffusion of the delivery plume. Corresponding simulations show that velocity gradients rapidly decay outside the immediate outlet vicinity, limiting convective spreading and preserving localized exposure. In μFPocket-2 (Fig. 3f–g) and μFPocket-5 (Fig. 3m–q), terminal and top outlets are optimized for directional delivery normal to the interface, producing compact concentration iso-surfaces that remain spatially confined. These results demonstrate that outlet geometry serves as a key design parameter for tuning both the directionality and spatial footprint of chemical stimulation. Beyond static outlet geometries, the μFPocket platform supports dynamic delivery modes based on coordinated use of multiple channels (Fig. 3i–k, **Fig. S23**). Multiple drugs can be delivered into a single confined region with precisely controlled concentration gradients (Fig. 3k). In particular, the delivery–removal configuration, in which one channel delivers drug while an adjacent channel simultaneously withdraws fluid, creates a localized recirculation zone that sharply confines the stimulated region. Provided with appropriate pressure control, the fluid field shows high stability during the observation (**Fig. S23e**). Optical experiments (**Supplementary Movie 7**) and simulations show that this push–pull mode dramatically suppresses lateral diffusion, enabling chemical stimulation areas as small as ~100 × 80 μm^2^, approaching single-neuron cluster dimensions. Alternative modes, including dual-channel co-delivery and asymmetric flow-rate control, further allow multiplexed or gradient-based stimulation using different compounds (**Fig. S23d**). Meanwhile, the design also allows us to control the temporal drug delivery patterns, supporting both burst and continuous release (**Fig. S23f**). To further elucidate directional chemical transport at the microfluidic–organoid interface, we performed a simplified numerical simulation to analyze the local flow field under representative delivery modes (**Fig. S24**). When the top-emitting outlet is positioned near the organoid surface, two distinct jet configurations emerge due to geometric asymmetry: a confined jet propagating along the channel direction (XZ plane) and a free jet oriented perpendicular to the channel direction (YZ plane). Consequently, the advective transport of chemical species exhibits a pronounced directional preference, governed by the spatial distribution of the velocity field and entrainment between the primary jet and the surrounding fluid. In conjunction with the outlet geometries and delivery modes presented in **Figs. S22–S23**, these results establish a coherent physical framework in which μFPocket-based delivery enables spatially selective surface modulation while maintaining a chemically stable global culture environment.

To analyze the performance of μFPocket in spatiotemporal chemical delivery for organoid stimulation, we tested μFPocket-1 with live brain cortical organoids under Ca^2+^ imaging. Fig. 4a–d directly compares conventional pipette-based conventional glutamate delivery with μFPocket-based glutamate delivery, highlighting the advantages of localized, time-locked chemical control in 3D neural organoids. Human cortical organoids (hCOs) expressing GCaMP8s used in this study were generated following a standard differentiation protocol (Fig. 4a). The hCO was placed in μFPocket-1 after sterilization, which is compatible with the imaging in a CO2-controlled microscope chamber (**Fig. S25**). We first recorded the neural activity by Ca^2+^ imaging under the baseline condition (no stimulation) or following bulk glutamate addition (50 μM, 40 μL) to the media using a conventional pipette (Fig. 4b, e–f, **Supplementary Movies 8–9**). Two representative regions of interest (ROIs 1–2, Fig. 4c) are selected for direct spatial comparison between local and distal responses. Under baseline conditions, neural activity remained sparse and spatially heterogeneous, reflecting spontaneous network dynamics. Conventional pipette-based glutamate delivery elicited Ca^2+^ responses across the organoid, with limited spatial specificity and substantial trial-to-trial variability, consistent with rapid dilution and uncontrolled diffusion in the surrounding medium. The strong fluidic flow caused by the pipette-based glutamate addition significantly moved the organoid, generating motion artifact in the optical recording. Based on our μFPocket, highly localized (~ 400 μm * 400 μm) glutamate delivery with controlled dosing can be realized, as shown in Fig. 4d. We also validate drug delivery feature of μFPocket-1 in a multistage site-specific glutamate delivery experiment (Fig. 4e–f). Using the same organoid, we selected 4 ROIs to guarantee consistency of comparison, as shown in Fig. 4e. In contrast to conventional chemical delivery, μFPocket-1 enables precise, surface-localized, and temporally defined delivery of glutamate to the same organoid. Using programmed pressure pulses applied through a single microfluidic channel (outlet located in ROIs 2–4), glutamate exposure is confined to a well-defined region on the organoid surface, as verified by the co-registered fluorescence of the glutamate tracer (cyan) and GCaMP8s signals (green) (Fig. 4e). Each ROI from 1–4 contains a microfluidic outlet, while ROI 1 was used as a control comparison without glutamate delivered. We sequentially apply a pulse-like delivery from the channels in ROIs 2–4, labeled as Stages 1–3. This is realized by an operation program using the syringe pump, as shown in Fig. 4f (blue, forward pumping ~ 5 s; gray, standby ~ 10 s; red, backward withdrawing ~ 5 s). Clear and sharp fluorescence signals of glutamate and GCaMP8s can be observed, with GCaMP8s signals consistently coming after glutamate. Meanwhile, the control region (ROI 1) shows no significant increase in both glutamate and GCaMP8s channels, proving the leakage-free feature of μFPocket. During Stages 2–3, correlation of fluorescence activities in ROIs 3–4 can be observed, due to partial overlapping of glutamate diffusion and network connectivity. Corresponding ROI-specific studies of baseline activity and conventional pipette-based delivery are presented in **Fig. S26**.

Beyond site-specific, highly localized glutamate delivery, μFPocket-1 enables multiple drug delivery modes with adjustable temporal profiles (Fig. 5, **Supplementary Movie 10**). To demonstrate this capability, we compare pulse-like and continuous glutamate release using the same organoid and the same active microfluidic channel, thereby minimizing differences in neural responses and channel variations. Four regions of interest (ROIs 1–4) are selected to evaluate spatially resolved activity, including three regions within or adjacent to the stimulated area (ROIs 1–3) and one control region (ROI 4) right next to the glutamate delivered area (Fig. 5a). Under baseline conditions, spontaneous Ca^2+^ activity remains low without coordinated activation across ROIs.

During pulse-like delivery, brief pump actuation generates rapid and transient glutamate exposure at the outlet, followed by active withdrawal to accelerate local clearance (Fig. 5b–c). This mode elicits sharply time-locked Ca^2+^ transients in ROIs located within the stimulated region, with fast onset and decay that closely tracked the delivery sequence. Repeated pulses create reproducible responses across stimulation cycles, demonstrating precise temporal control over chemical input and neural activation. The operation program of the syringe pump is shown in Fig. 5c by colored bars (blue, forward pumping ~ 5 s; gray, standby ~ 5 s; red, backward withdrawing ~ 5 s). In contrast, continuous delivery (forward pumping ~ 5 s; standby for the rest of recording) generates a gradual accumulation of glutamate near the outlet and sustained elevation of GCaMP8s signals in responsive ROIs. Rather than discrete transient, neural activity evolves into prolonged and temporally integrated responses (showing a saturation after 30 s), consistent with persistent receptor activation under maintained chemical exposure. Importantly, the magnitude and kinetics of responses varied across ROIs, reflecting both the local concentration field and the intrinsic functional heterogeneity of the organoid. The region closest to the outlet (ROI 3) exhibits the strongest and earliest activation, whereas the more distal ROI 2 showed attenuated or delayed responses. The control region (ROI 4) remained minimally responsive under both delivery modes, confirming that stimulation remained spatially confined despite operation in open liquid media. While both ROIs 2–3 show responses in GCaMP8s activities under continuous mode, ROI 2 remains silent in both glutamate and GCaMP8s channels using pulse-like delivery. This highlights the feature not only in spatial resolution but also in higher dosing precision.

Taken together, these results establish μFPocket as a platform that provides orthogonal and programmable control over both the spatial location and temporal profile of chemical delivery, enabling localized interrogation of neural circuits within intact 3D organoids at both regional and even single-cell resolution. In contrast to conventional bulk or pipette-based delivery, which couples chemical exposure to uncontrolled diffusion and mechanical perturbation, μFPocket-based stimulation confines drug input to defined surface sites and time windows. This confinement yields orders-of-magnitude improvements in spatiotemporal precision, allowing effective neural activation with minimal drug volume while preserving the global chemical and mechanical environment of the culture. By decoupling chemical input from bulk diffusion and fluidic disturbance, the μFPocket enables direct, causal mapping between localized pharmacological perturbations and neural activity dynamics in complex 3D tissues, an essential capability for mechanistic studies of circuit function and pharmacological modulation.

To illustrate the multimodal capability of the integrated μEFPocket, we carried out the electrical stimulation experiments, as shown in Fig. 6. We achieved simultaneous manipulation and monitoring of neural activity in hCOs. We use the μEFPocket-3 to interface with the organoid while maintaining optical transparency, allowing wide-field Ca^2+^ imaging across the tissue (Fig. 6a). Representative fluorescence images following electrical stimulation reveal robust activation of neuronal populations distributed across the field of view, confirming that the integrated interface preserves both electrical functionality and high-quality optical access. The spatial programmability of electrical stimulation was investigated by comparing global and localized stimulation configurations (Fig. 6b–d). In the global mode, all electrodes distributed across the interface were activated simultaneously, whereas in the local mode, only a small subset of electrodes were driven. Ca^2+^ imaging reveals that these two configurations produce markedly different spatial activation patterns. Global stimulation induces widespread neuronal responses throughout the organoid, while localized stimulation generates spatially confined activity originating near the stimulated electrodes (Fig. 6b). Quantitative latency analysis further confirms that neurons located closer to the stimulation site respond earlier than distal regions (Fig. 6c), indicating that the μEFPocket platform enables controlled spatial modulation of neural activation. Latency plots based on fluorescence activity (Fig. 6d) are provided to better illustrate the network activity propagation through neurons. Time-resolved imaging further reveals the propagation dynamics of network activity following electrical stimulation (Fig. 6e). Upon stimulation after 20 s, Ca^2+^ signals first appear near the stimulation region and subsequently spread outward as propagating wavefronts across the neural network, highlighted by the expanding red dashed circles. This observation indicates that electrical perturbations introduced through the integrated interface can trigger coordinated network-level responses within the organoid, enabling investigation of signal propagation and connectivity in three-dimensional neural tissues. The corresponding fluorescence activity data with stimulation timestamps are presented in **Fig. S27–28** (global mode) and **Fig. S29–30** (local mode). Clear response of neural activity can be observed in the 3–4 rounds of electrical stimulation (**Supplementary Movies 11–12**).

To further demonstrate multimodal recording capability, synchronized electrical and optical measurements were performed during repeated stimulation cycles (Fig. 6f–j). The applied stimulation pattern is shown in Fig. 6f, with timestamp labeled by red lines. Ca^2+^ fluorescence traces extracted from regions of interest located near stimulating electrodes (Fig. 6g) show reproducible responses following each stimulation event, as reflected in the corresponding spectrograms. The ROIs near electrodes are single neurons responding to the stimulation. Concurrently, extracellular electrical recordings reveal characteristic spike waveforms associated with neuronal firing (Fig. 6j). Corresponding spike analyses are demonstrated in **Fig. S31**. The temporal alignment between optical signals and electrophysiological activity confirms that the MEA–μFPocket platform enables simultaneous electrical stimulation, electrical recording, and optical monitoring within the same device. We also study the neural activity response from different conditions of electrical stimulation, as shown in **Fig. S32**, finding the preferable condition of stimulation frequency (±50 μA, 5 ms / phase at 20 Hz). Together, these results highlight the ability of the μFPocket architecture to support integrated multimodal interrogation of neural activity in three-dimensional organoid systems.

## Conclusion

In this study, we introduce microElectroFluidicPocket (μEFPocket), a family of multimodal in vitro biointerfaces that enable simultaneous chemical, electrical, and optical communication with intact three-dimensional tissues. Built on an ultrathin, highly biocompatible Parylene C (PPX-C) matrix, the platform combines monolithically integrated microfluidics, bioelectronics, and mechanically programmed architectures within a single wafer-scale fabrication framework, providing a scalable route to tissue-like interfaces that are mechanically compliant, optically accessible, and functionally versatile. In contrast to conventional microfluidic approaches based on thicker bonded structures such as PDMS or rigid photoresists, PPX-C deposition enables fully enclosed, micrometer-scale thin-film microchannels and dense multilayer integration while preserving extreme flexibility and conformal contact. Functionally, μEFPockets overcome the long-standing separation between localized chemical perturbation and functional readout in organoid systems by enabling highly localized and temporally programmable drug delivery together with electrophysiological stimulation/recording and real-time optical imaging. This capability creates new opportunities for simultaneous multimodal interrogation, including multiplexed drug–electrical stimulation, region-specific functional mapping, spatially patterned perturbation of heterogeneous or mixed-lineage organoids, and closed-loop studies of tissue responses. More broadly, μEFPockets establish a versatile and scalable framework for site-specific interaction with complex 3D biological systems, with broad relevance to organoid-based neuroscience, disease modeling, pharmacological screening, and next-generation in vitro therapeutic testing.

## Methods

### Materials and devices

#### Materials.

Silicon wafers (<100> orientation, single side polished, test grade) were obtained from University Wafer. Dichloro-[2,2]-paracyclophane (DPX-C, Parylene-C dimer) was used for the chemical vapor deposition (CVD) of Parylene-C (PPX-C) and was obtained from Specialty Coating Systems. AZ 10XT (520 cP) was used as the positive photoresist to prepare the sacrificial microfluidic (μF) channel template and was obtained from MicroChemicals. AZ 400K (1:3) was used to develop the AZ 10XT pattern and was obtained from MicroChemicals. S1813 and AZ 300 MIF were used for preparing the etching mask of metal patterns and were obtained from Kayaku Advanced Materials and MicroChemicals. Hexamethyldisilazane (HMDS) was used as the adhesion promoter for photoresists and was obtained from MicroChemicals. Copper etchant CE-100, chromium etchant 1020, and gold etchant were obtained from Transene Inc. Dow Sylgard 184 silicone was used to fabricate the drug reservoirs and was obtained from Dow Corning. ARClear 92734 was used as the bonding adhesive between the drug reservoir and the PPX-C μFs and was obtained from Adhesive Research. Sil-Poxy rubber adhesive was used to strengthen the bonding between PPX-C and PDMS or silicone rubber and was obtained from Smooth-On Inc. Rhodamine B was used as a water-soluble pigment and obtained from Thermo Fisher Scientific. 1x Dulbecco’s phosphate buffered saline (DPBS) was used for electrode characterization and was obtained from Corning. Anisotropic conductive film (ACF) cable was used for electrode connection and was obtained from Shin-Etsu Polymer and Hitachi Chemical (AC-7813KM-25). Microfluidic tubing (Product no. DM-PTFE-0803–30, 1/32” OD × 0.3 mm ID) and fitting (Male Mini Luer Fluid Connectors, 0.8–1 mm ID) were purchased from Darwin Microfluidics.

All materials used in the fabrication are listed above unless otherwise mentioned.

### Fabrication of PPX-C based microfluidics.

#### Fabrication of microfluidics only^54–57^.

The general method of PPX-C based microfluidics fabrication corresponds to **Figure S7**. Thickness of layers (PPX-C and template) can be varied. (1) A 5-μm-thick PPX-C layer was coated by CVD on the silicon oxide layer of a 500-μm-thick silicon wafer. The polished side was used for following fabrication. (2) A layer of AZ 10XT was uniformly spin coated on the PPX-C, at 800–1000 r.p.m. to obtain a thickness ranging from 12 μm to 14 μm. This is followed by a soft baking at 110 °C for 3 min and a 30–60 min rehydration. (3) The spin-coated AZ 10XT was patterned with UV light (500 mJ cm^−2^) and developed in AZ 400K (1:3). (4) A hard baking at 110 °C for 1 min to anneal the surface of AZ 10XT positive template. (5) The PPX-C surface with AZ 10XT patterns was cleaned and activated by O_2_ plasma treatment for 30 s to enhance bonding. (6) A 5-μm-thick top PPX-C layer was coated by CVD on the patterned side of the existing bottom PPX-C. (7) A 100-nm Cu layer was deposited on the bottom PPX-C via electron-beam evaporation. (8) A 14-μm-thick AZ 10XT layer was spin-coated on the top PPX-C and patterned following Step (2)-(3). Alignment was needed. (9) The exposed Cu was etched by copper etchant CE-100 (1:1 diluted with water) to prepare the etching mask for PPX-C. (10) The unmasked PPX-C was removed by the reactive ion etching (RIE) using O_2_. Top μF channel outlets and μF connectors were opened simultaneously. (11) The RIE mask (AZ 10XT and Cu) was removed by acetone and CE-100 solution. (12) The prepared μF devices were released from wafer via water rinsing and then soaked in acetone for 12–24 hours to completely remove the sacrificial AZ 10XT template (**Supplementary Movie 1**).

#### Fabrication of MEA-integrated microfluidics.

This corresponds to **Figure S11**. Thickness of layers (PPX-C, metal, and template) can be varied. (1) A PPX-C layer (2–5 μm) was coated by CVD on the silicon oxide layer of a 500-μm-thick silicon wafer. (2) A 20-nm-thick Cr film and a 200-nm-thick Au film were sequentially deposited via electron-beam evaporation. (3) An HMDS adhesion layer was spin-coated at 3000 r.p.m., then followed by a 3-min baking at 110 °C. (4) A layer of S1813 was spin-coated at 3000 r.p.m., then followed by a 1-min soft baking at 110 °C. (5) The S1813 layer was patterned with UV light (50 mJ cm^−2^) and developed in AZ 300 MIF (1:3). (6) The exposed metal was etched by Au etchant and Cr etchant sequentially. Upon completion of the etching, the wafer was rinsed by acetone to remove photoresist. (7) The PPX-C surface with metal patterns was cleaned and activated by O_2_ plasma treatment for 30 s to enhance bonding. (8) A PPX-C layer (2–5 μm) was coated by CVD to encapsulate the metal layer. (9) The monolithic preparation of μF layers followed Step (2)-(12) of the Fabrication of microfluidics only section. Alignment was required for each step of exposure.

#### Fabrication of the drug reservoir^58^.

(1) A piece of ARClear 92734 adhesive was fixed to the bottom of a petri dish with the release liner on the top side removed. (2) PDMS base and its curing agent were mixed (10:1) and poured onto the petri dish with ARClear adhesive, then degassed under vacuum for 2 min and left for curing at 60 °C for 12 hours. (3) The cured ARClear-PDMS was obtained from the petri dish and cut by a razor blade to define the outline of the reservoir. (4) The ARClear release liner on the other side was patterned to label the positions of reservoir through holes and center pocket opening. Through holes for fluidic tubing and fitting were cut by a biopsy punch with a diameter of 2.5 mm. The center opening for μFPockets was cut by a biopsy punch with a diameter of 6–10 mm. The release liner was kept on the surface until device assembly.

### Fabrication of 3D microfluidic devices.

#### Fabrication of μFTest-2 and μEFTest-1.

2D precursors of μFTest-2 and μEFTest-1 were prepared following Fabrication of PPX-C based microfluidics section. The 2D precursors were carefully transferred by a cover slip and kept on it. The thin terminals of both devices were designed as fluidic connectors and directly inserted into tubes. Two terminals can be inserted into the same tube or separated tubes. This is followed by UV glue sealing of tube openings to ensure encapsulation. Then, the connected test devices can be fitted into the catheter (diameter ~ 3 mm) and delivered.

#### Fabrication of μFPocket-1.

This corresponds to **Figure S3**. 2D precursors of μFPocket-1 were prepared following Fabrication of microfluidics only section. (1) An acrylic template film was processed by UV laser to label the bonding sites and then was used to transfer the 2D precursor from water. (2) The 2D precursor was carefully folded according to the bonding labels to form a 3D pocket structure. (3) Then the folded structure was transferred to the adhesive surface of reservoir with connectors aligned. (4) The other side of the pocket was encapsulated by another media reservoir with a hole (6–10 mm) in the middle. (5) The bottom of the media reservoir was sealed with a cover slip (1.5-mm thickness) by plasma bonding.

#### Fabrication of μEFPockets.

Electronics integration was achieved by bonding the contact pads to custom-designed flexible printed circuits (FPCs, JLCPCB, China). An anisotropic conductive film (ACF, AC-7813KM, Hitachi Ltd., Japan) was used to provide vertical electrical conduction while maintaining lateral insulation between adjacent pads. Fluidic connections were the same as ***Fabrication of μFPocket-1***.

#### Electrochemical Deposition of Platinum Black and Impedance Characterization^25,59^.

Platinum black (PtB) was electrodeposited onto the Au microelectrodes by chronoamperometry (CA) using a three-electrode configuration (SP-50e, BioLogic, France). A platinum wire served as the counter electrode and an Ag/AgCl electrode as the reference electrode. Deposition was carried out at −0.1 V for 20 s in a solution containing 3 wt% chloroplatinic acid (Sigma-Aldrich, USA) and 0.1 wt% lead acetate (Sigma-Aldrich, USA). After deposition, the electrodes were rinsed with deionized (DI) water.

Electrochemical impedance spectroscopy (EIS) measurements were performed in phosphate-buffered saline (PBS, pH 7.4, Sigma-Aldrich, USA) over a frequency range of 10 Hz to 100 kHz using the same potentiostat system (SP-50e, BioLogic, France).

### Finite element analyses (FEA).

#### Solid structure.

Three-dimensional (3D) finite element analyses (FEA) were conducted using the commercial software package Abaqus to capture the post-buckling behavior of the two-dimensional (2D) precursor structures. The precursors were modeled using four-node shell elements, and composite layups were employed when metallic trace layers were present. Linear buckling analyses were first performed under compressive loading to determine the critical buckling strain and the associated buckling mode for each loading condition. The obtained buckling modes were subsequently used to guide post-buckling deformation analysis. Parylene-C was modeled as a linear elastic material with a Young’s modulus of *E*_Parylene-C_ = 2.76 GPa and a Poisson’s ratio *ν*_Parylene-C_ = 0.4. Gold (Au) was described using an idealized elastoplastic constitutive model without strain hardening, with an elastic modulus and Poisson’s ratio of ^*E*^_Au_ = 79 GPa and ^*ν*^_Au_ = 0.4.

#### Fluid.

The commercial software COMSOL Multiphysics was employed to numerically characterize the fluid flow field and the transient transport behavior of dissolved species. The model coupled the Laminar Flow and Transport of Diluted Species physics interfaces. Water was assumed as the working fluid with standard thermophysical properties, and the diffusion coefficient of Rhodamine B (RhoB) at room temperature was set to *D*_RhoB_ = 3.6e^−10^ m^2^/s. The computational domain was discretized using tetrahedral elements, with local mesh refinement applied near the channel outlet to capture concentration gradients and boundary-layer refinement imposed along the channel walls to accurately resolve near-wall flow behavior. A fully developed velocity profile was prescribed at the channel inlet. The evolution of the concentration field was visualized using iso-surfaces corresponding to the inlet concentration, enabling clear identification of species propagation within the flow.

### Dorsal forebrain human organoid culture method.

Organoids were derived following previously reported protocol that used combined small-molecules treatment to accelerate neuronal maturation with few modifications^60–62^.**1** Briefly, PGP1 iPSCs were maintained in mTeSR+ on Matrigel-coated plates, passaged at least twice, and dissociated with Accutase before aggregation into embryoid bodies. Aggregation was initiated with 10,000 cells per well in 96-well ultra-low-attachment V-bottom plates containing mTeSR+ supplemented with Y-27632. Neural induction was performed from day 1 to day 8. From day 1 to day 8, medium was changed every 2 d with Essential 6 medium supplemented with 10 μM SB431542, 100 nM LDN193189 and 2 μM XAV-939. On day 8, the medium was switched to organoid growth medium consisting of a 50:50 mixture of Neurobasal and DMEM/F12 medium with 1% B27, 0.5% N2, 1% GlutaMAX, 0.5% minimal essential medium (MEM) nonessential amino acid (NEAA) solution, 0.1% 2-mercaptoethanol. On day 18, organoids were transferred to 10-cm dishes at roughly 20 organoids per dish and placed on an orbital shaker set to gentle motion to prevent organoid fusion. Small molecule cocktail was composed of four small molecules—GSK2879552, EPZ-5676, Bay K 8644 and NMDA (MedChemExpress) applied at a working concentration of 1 μM each and applied from day 18 to day 35. At day 40, cortical organoids were transduced with a lentiviral vector encoding GCaMP8s under the human Syn1 promoter to achieve neuron-specific expression. Lentiviral constructs were custom-designed and packaged by VectorBuilder (Chicago, IL, USA). Viral particles (1 × 10^9^ TU/mL) were applied for 24hr. at a multiplicity of infection (MOI) of 10. After 7 days post transduction organoids were used for calcium imaging experiments.

### In situ Ca^2+^ imaging.

For calcium imaging experiments, GCaMP8s-expressing cortical organoids were placed on a sterilized mesh support device to maintain stability during imaging. Devices were sterilized in 70% ethanol for 10 minutes, followed by three washes in 1× PBS prior to use. Organoids were imaged in BrainPhys^™^ Imaging Optimized Medium (STEMCELL Technologies, Vancouver, Canada). Calcium imaging was conducted using an Olympus Corporation FV3000 Fluoview confocal microscope equipped with a 4X objective. Time-lapse images were acquired as single optical planes at 15.2 frames per second. GCaMP8s fluorescence was excited using a 488 nm laser. Glutamate delivery was visualized using Alexa Fluor^™^ 647 dye (Thermo Fisher Scientific) and detected using 657 nm excitation. Imaging parameters, including laser power, detector gain, and acquisition settings, were maintained constant across experimental conditions. L-Glutamic acid monosodium salt (Sigma-Aldrich, St. Louis, MO, USA) was applied at a final concentration of 50 μM to induce calcium responses.

### Calcium Imaging processing and analysis

Multi-region fluorescence measurements were extracted from Fiji (ImageJ) using the multi-measure function. Regions of interest (ROIs) were manually defined across spatially distinct areas of each organoid based on their location relative to mesh-based delivery sites (inside vs. outside delivery regions). Mean fluorescence intensity values were exported as CSV files for downstream analysis. Quantitative analysis was performed Data analysis and visualization were performed in R (version 4.3.1; R Foundation for Statistical Computing, Vienna, Austria). Single cell fluorescence analysis was done with suite2p package (version 0.14.6) implementing cellpose (version 4.0.7) for segmentation. Cell body localized fluorescence signal was exported to Python (version 3.9.23) for analysis. Frame numbers were converted to time (seconds) using the acquisition rate (15.2 frames per second). Baseline fluorescence (F_0_) was defined as the mean fluorescence intensity across frames preceding the first stimulation event.

Normalized calcium responses were calculated as ΔF/F_0_, defined as (F – F_0_) / F_0_, where F represents fluorescence intensity at time *t* and F_0_ represents baseline fluorescence. ΔF/F_0_ traces were computed independently for each ROI. Time-series traces were visualized using ggplot2 package (version 3.5.1) or matplotlib (version 3.9.4). To assess spatial response patterns across ROIs, ΔF/F_0_ values were reshaped into long format and displayed as heatmaps using the viridis “inferno” color scale. Individual ROI traces were additionally plotted separately to evaluate single-region response dynamics.

### Electrophysiological Recording and Stimulation

Electrophysiological recordings were performed using a 128-channel RHS stimulation/recording system (Intan Technologies, USA). The device was connected to the headstage through a custom-designed printed circuit board. Signals were acquired at a sampling rate of 30 kHz and recorded using Intan RHX software. Upon acquiring signal, a 300 to 6000Hz Butterworth bandpass filter was implemented via Spikeinterface (version 0.103.0). A sliding-window standard deviation was computed for each channel using a 1-second (30,000-sample) window via FFT-based convolution. Putative spikes were detected as peaks in the absolute value of the mean-subtracted filtered signal that exceeded 5× the local standard deviation, with a minimum inter-peak distance of 80 samples (~2.67 ms). For each detected peak, a waveform snippet of 80 samples was extracted (40 samples pre-peak, 40 samples post-peak, corresponding to ±1.33 ms). Snippets at recording boundaries or with incomplete waveforms were discarded. Spike waveforms were projected into a two-dimensional space using principal component analysis (PCA). K-means clustering was applied to the PCA projections, with the optimal number of clusters determined via the elbow method. Mean waveform templates with standard deviation envelopes and full width at half maximum (FWHM) were computed per cluster and visualized using matplotlib (version 3.9.4). Electrical stimulation was delivered in a current-controlled mode using charge-balanced biphasic pulses. Each stimulation train consisted of five pulses, with current amplitudes ranging from 10 to 50 μA and pulse frequencies of 5–100 Hz. Each pulse had a total duration of 10 ms, composed of two phases (negative and positive in sequence) of 5 ms each. Multiple stimulation was delivered at 30 s intervals to allow sufficient recovery between trials. To remove stimulation artifacts from all recording channels, raw data within each stimulation window — including 500 ms before and after the stimulation sequence — is replaced by linear interpolation between the boundary values. Spikes detected within these windows are excluded from further analysis.

## Supplementary Material

Supplementary Files

This is a list of supplementary files associated with this preprint. Click to download.

- SupplementaryMaterials.pdf
- SupplementaryMovie8DeliveryComparison.mp4
- SupplementaryMovie12ComparisonofDifferentElectri.mp4
- SupplementaryMovie11NeuralResponsesuponElectricalStimulation.mp4
- SupplementaryMovie7DeliveringModesDemonstration.mp4
- SupplementaryMovie6MediaRefreshing.mp4
- SupplementaryMovie10MultimodeGlutamateDelivery.mp4
- SupplementaryMovie3XXFPocket1SolutionTest.mp4
- SupplementaryMovie1ChannelOpening.mp4
- SupplementaryMovie5XXEFTest1andXXFTest2Demo.mp4
- SupplementaryMovie2XXFPocket1SequentialReleasing.mp4
- SupplementaryMovie4XXEFPocket1SolutionTest.mp4
- SupplementaryMovie9SitespecificGlutamate.mp4

## Figures and Tables

**Figure 1:**
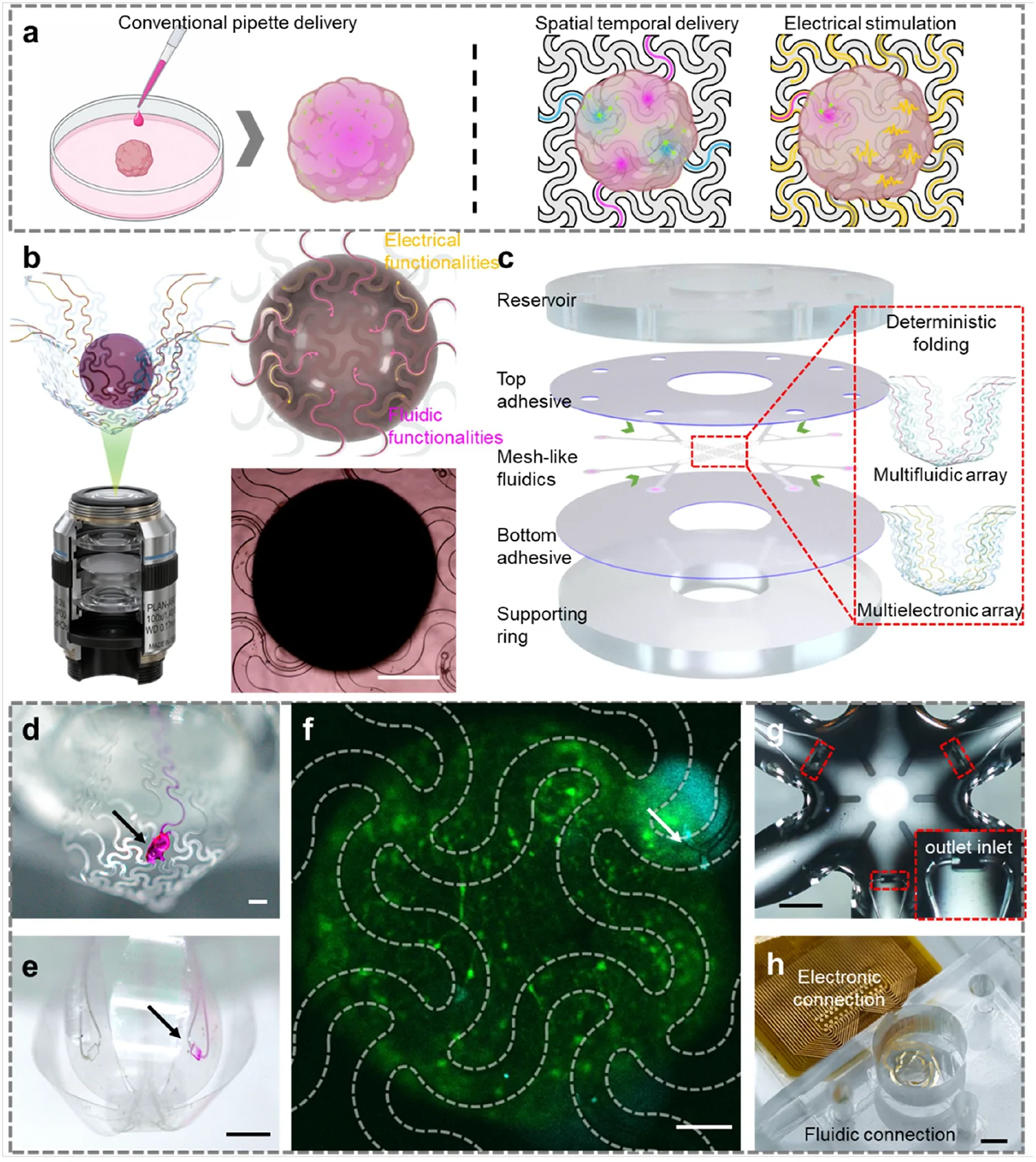
3D μEFPocket for multimodal communication with organoids. **a.** Schematic comparison between conventional pipette-based and μEFPocket-based drug delivery. The μEFPocket enables spatial-temporal control of drug delivery, electrical stimulation and recording, and calcium imaging, all operated in a simultaneous fashion. **b.** 3D schematic illustration of μEFPocket holding an organoid with integrated electrical-fluidic interfaces and real-time optical accessibility. Optical microscopy showing an organoid in the μFPocket is presented. **c.** Exploded view of an μFPocket. **d-e.** Optical images of two configurations, named as μFPocket −1 and μFPocket-4, with one channel delivering dye solutions. **f.** In situ fluorescence imaging using an μEFPocket holding a human cortical organoid to record neural activities (green) and glutamate release (blue). **g.-h.** Optical images of a conformal μFPocket (**g**) and an μEFPocket (**h**). Scale bars: 500 μm in **b**, **d**, **e**, and **g**; 200 μm in **f**, 2 mm in **h**. demonstrates the concept, architecture, and functional capabilities of the μFPocket platform for localized chemical stimulation of 3D neural organoids with simultaneous optical readout. Conventional pipette-based drug delivery (Fig. 1a, left) introduces substantial spatial and temporal uncertainty due to diffusion, fluid convection, and mechanical disturbance of the tissue, which limits reproducibility and precludes precise correlation between stimulus timing, spatial location, and cellular response. In contrast, the μFPocket enables confined, time-locked, and minimally invasive delivery of chemical stimuli directly to the organoid surface while preserving multimodal access for either electrical stimulation or optical imaging (Fig. 1a, right). This integrated approach addresses a key technical limitation in studying localized pharmacological modulation in 3D tissue models.

**Figure 2:**
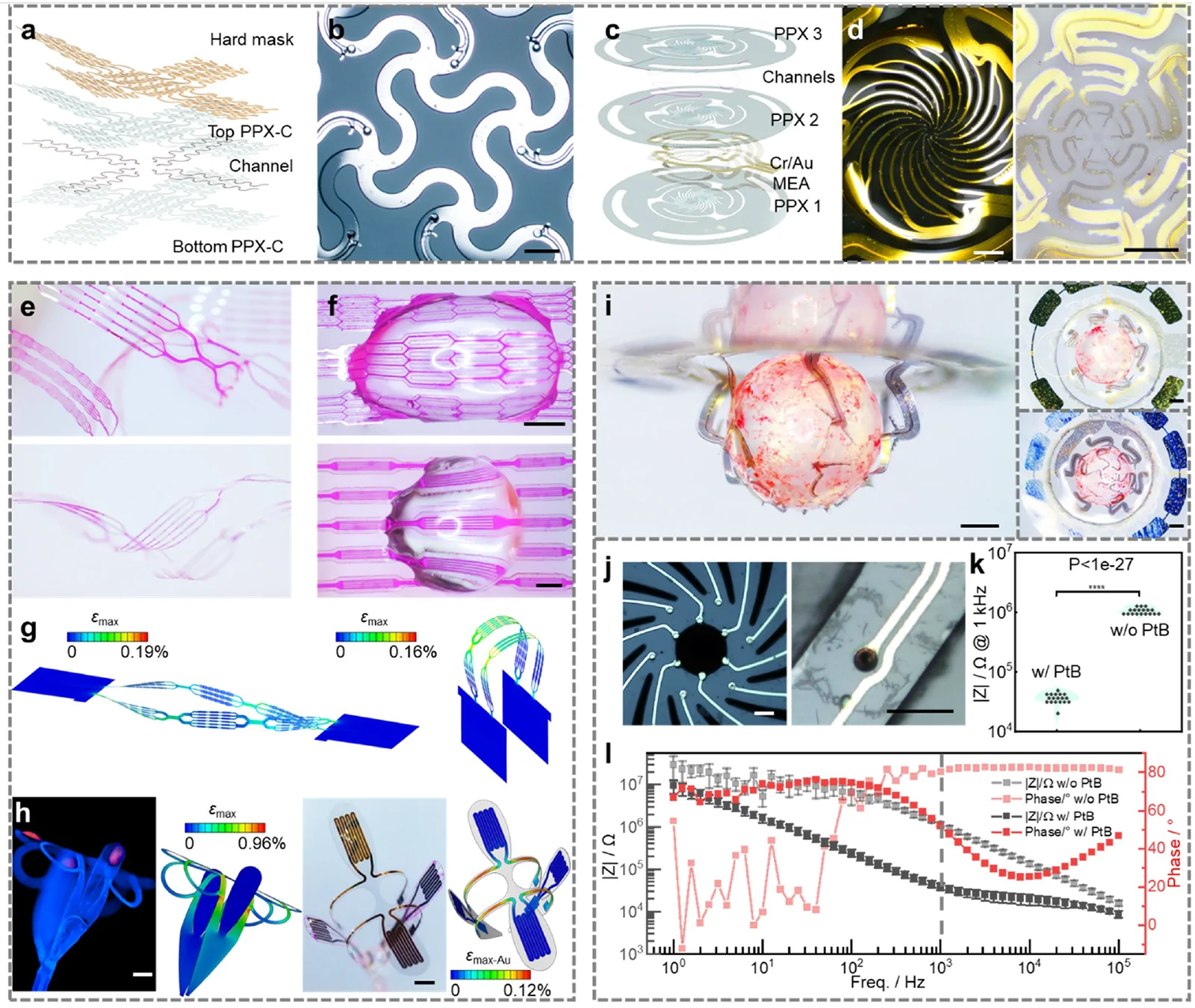
Structural design and mechanical integrity of 3D μEFPockets. **a.** Exploded view of the structural layout of a PPX-based microfluidics. **b.** Optical image of an μFPocket-1, highlighting multiple microfluidic channels and customized outlets. **c.** Exploded view of the fabrication of PPX-based microelectrofluidics. **d.** Optical image of fabricated μEFPockets (Left, μEFPocket-1; right, μEFPocket-2). **e.-f.** Mesh-like PPX-C microfluidics for mechanical testing (μFTest-1). The test device undergoes 360-degree twisting and 180-degree bending while maintaining mechanical integrity. **g.**The corresponding finite element analyses (FEAs) of μFTest-1 under twisting and bending. **h.** Rose-shape PPX-C microelectrofluidics showcasing mechanical compliance and corresponding FEAs. **i.** 3D extended structure of μEFPocket-2 with a sphere (diameter=2.1 mm). Upper-right, top view; lower-right, bottom view. **j.** Microelectrodes before (left) and after (right) PtB deposition. **k.-l.** Impedance comparison between MEA before and after PtB deposition. Grubb’s test (α=95%) was used to detect and remove outliers in the EIS analysis. T-test was used to compare MEA with and without Pt black (PtB) electroplating (p<1e-27). Scale bars: 250 μm in **b**; 500 μm in **d**, **f** and **i**; 1 mm in **h**; 50 μm in **j**.

**Figure 3:**
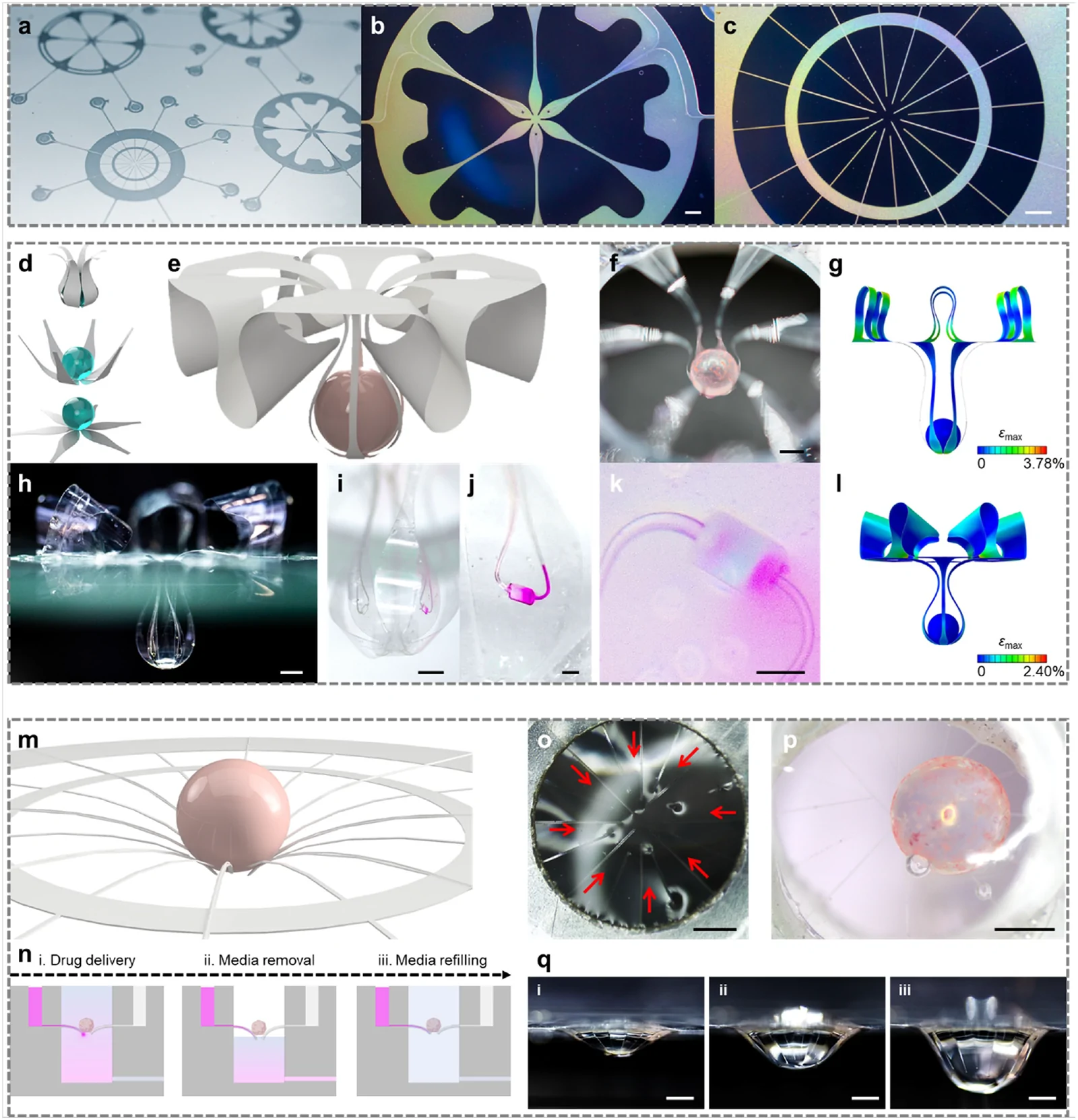
FEA-guided mechanical designs of highly conformal interfacing μFPockets. **a.-c.** General view of the manufacturability and planar structures of conformal μFPockets (named as μFPocket-2, 3, 4, 5) prior to their wafer releasing (**a**), μFPocket-2 (**b**) and 5 (**c**) on a wafer. **d.-e.** Schematic illustration of a conformal wrapping design strategy based on leaf-shaped segment. Two-dimensional design of the pocket section was based on 6-fold dividing of a sphere (**d**). Structure is maintained and stabilized by mechanical bonding of peripheral section (**e**). **f.-g.** The optical image (**f**) and FEA (**g**) of μFPocket-2 holding an organoid phantom (Ф=2 mm). **h.-l.** Structures of deterministically folded μFPocket-4 (**h**), its channel design (**i-k**), and FEA result (**l**). **m.** Schematic illustration of adaptive contacting strategy based on cantilever microfluidic probes. **n.** Schematic illustration of media refreshing capability of μFPocket-5 assembly. **o.-p.**Optical images of μFPocket-5 in contact with water (o) and with an organoid phantom (p, Ф=2 mm). Microfluidic channels are labeled by arrows. **q.** Structural flexibility of μFPocket-5. Water was added from the top to mimic liquid dragging force and to showcase the shape changing performance. Scale bars: 1 mm in **b**, **c**, **f**, **h**, **o-q**; 500 μm in **i**; 100 μm in **j**, **k**.

**Figure 4:**
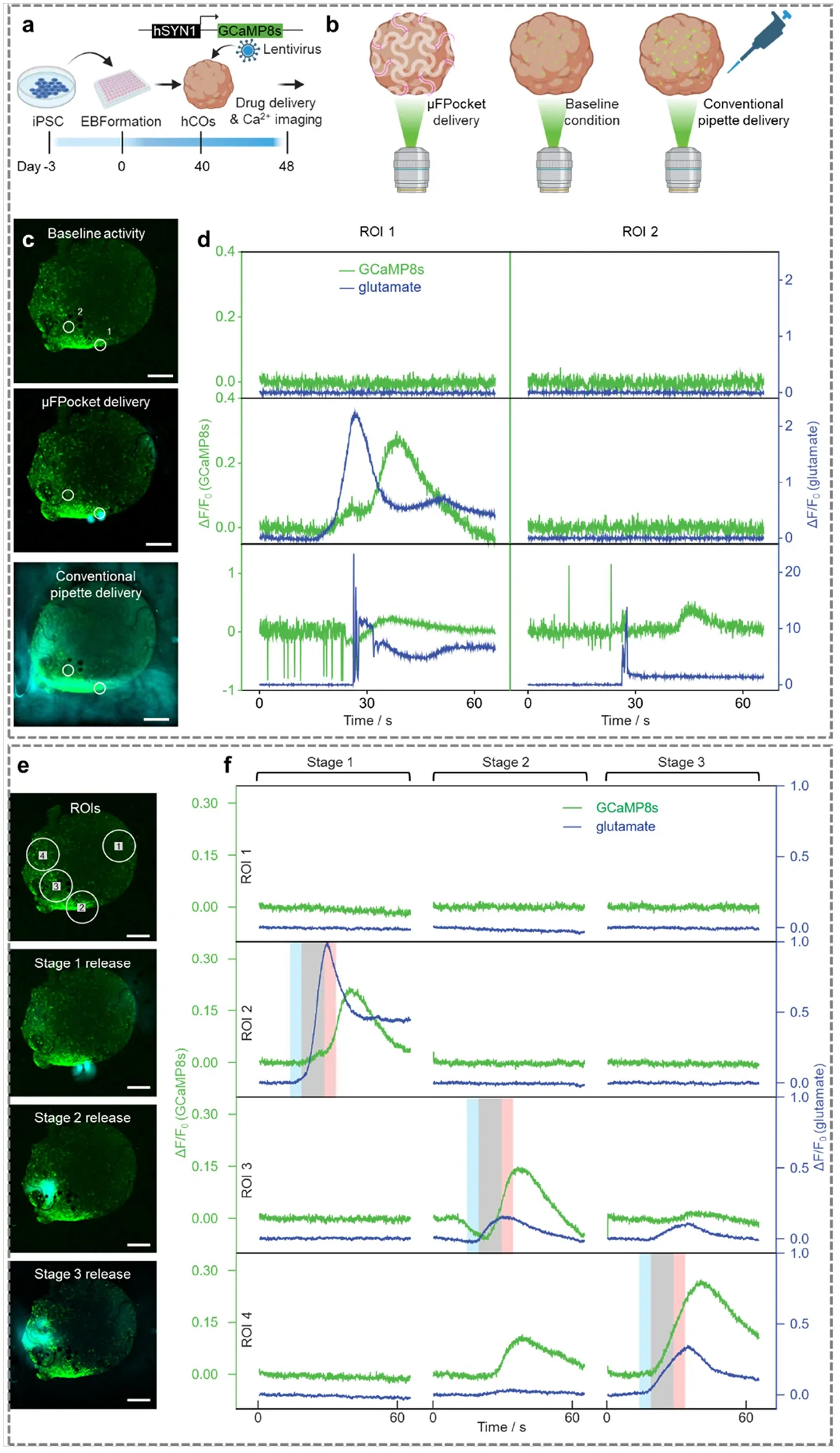
Spatiotemporal control of drug delivery and simultaneous calcium imaging with μFPockets. **a.-b.** Schematic illustrations of neural organoid culture protocol (**a**) and neural activity comparison between μFPocket drug delivery, baseline, and conventional pipette delivery by pipette (**b**). **c.-d.** Quantitative fluorescence analyses of neural activities of baseline condition (top, with ROIs labeled), glutamate delivery using μFPocket (middle), and conventional glutamate delivery using a pipette (bottom). We use a dashed line to label the time of pipette-based delivery of 40 μL glutamate. **c.** Fluorescence images showing baseline condition and conventional delivery of glutamate. Cyan, glutamate; green, GCaMP8s. **d.** ROI-specific fluorescence activity analyses corresponding to **c**. **e.-f.** Quantitative fluorescence analyses of neural activities of site-specific glutamate release using μFPocket. We selected four regions of interest (ROIs 1–4, shown in **e**) to study neural activity separately. **e.** Fluorescence images showing three sequential stages of site-specific glutamate release. **f.** Corresponding ROI-specific fluorescence activity analyses. Color bars indicate corresponding status of the pump control (blue, pumping; gray, standby; red, withdrawing). All the experiments demonstrated in this figure use the same organoid. Scale bars: 500 μm.

**Figure 5:**
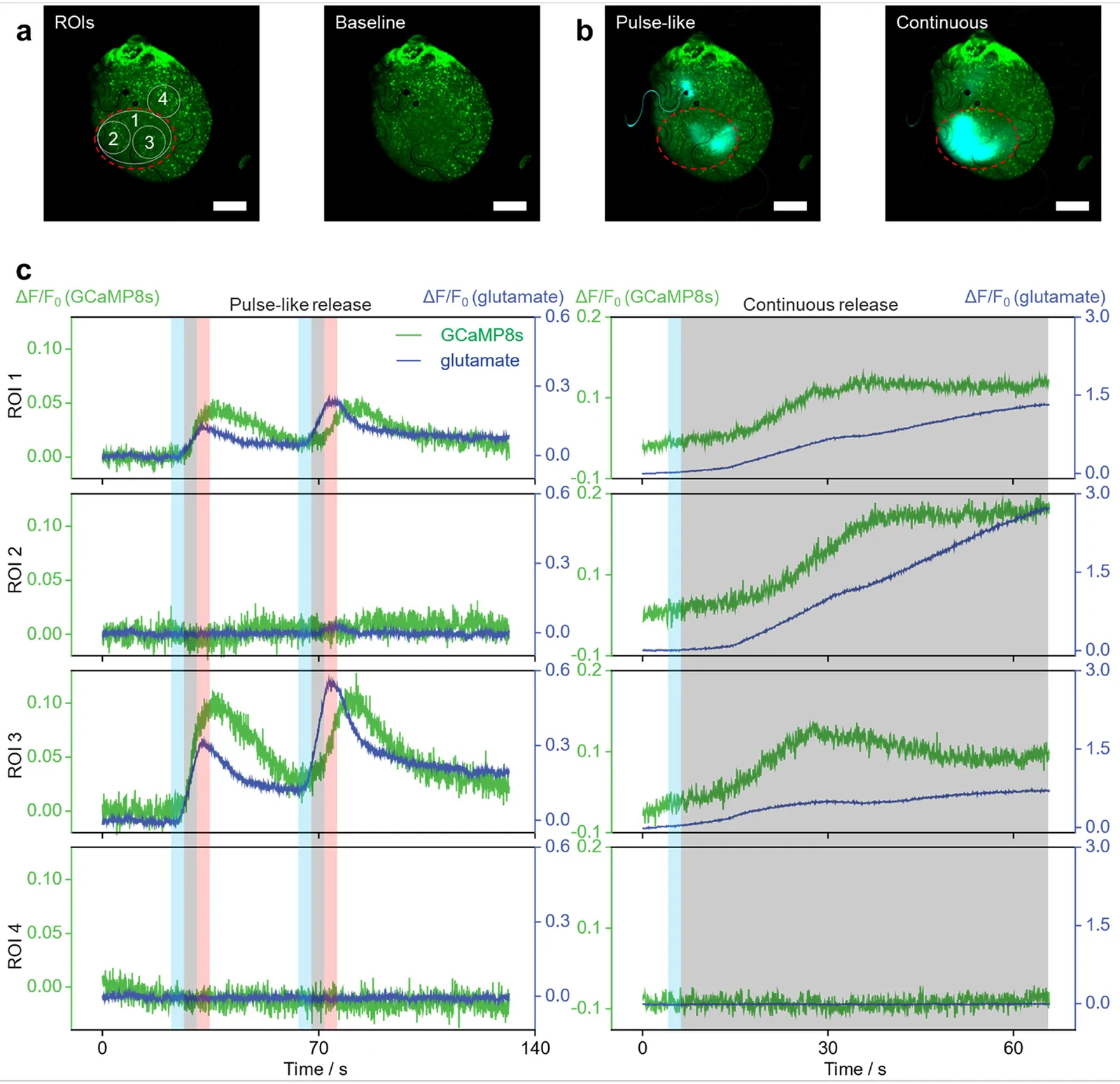
Tunability in drug releasing profile. **a.** Fluorescence images showing ROI selection and baseline condition activity. **b.** Fluorescence images showing pulse-like and continuous delivery of glutamate. Cyan, glutamate; green, GCaMP8s. **c.** Quantitative fluorescence analyses of neural activities of pulse-like and continuous glutamate delivery using μFPocket. Analysis of four selected regions of interest (ROIs 1–4, shown in **a**) on the neural activity reveals spatial localization and the variations within the glutamate delivered region (ROIs 1–3) and a control region (ROI 4). Color bars indicate corresponding status of the pump control (blue, pumping; gray, standby; red, withdrawing). All the experiments demonstrated in this figure use the same organoid, with the same active microfluidic channel delivering glutamate. Scale bars: 500 μm.

**Figure 6:**
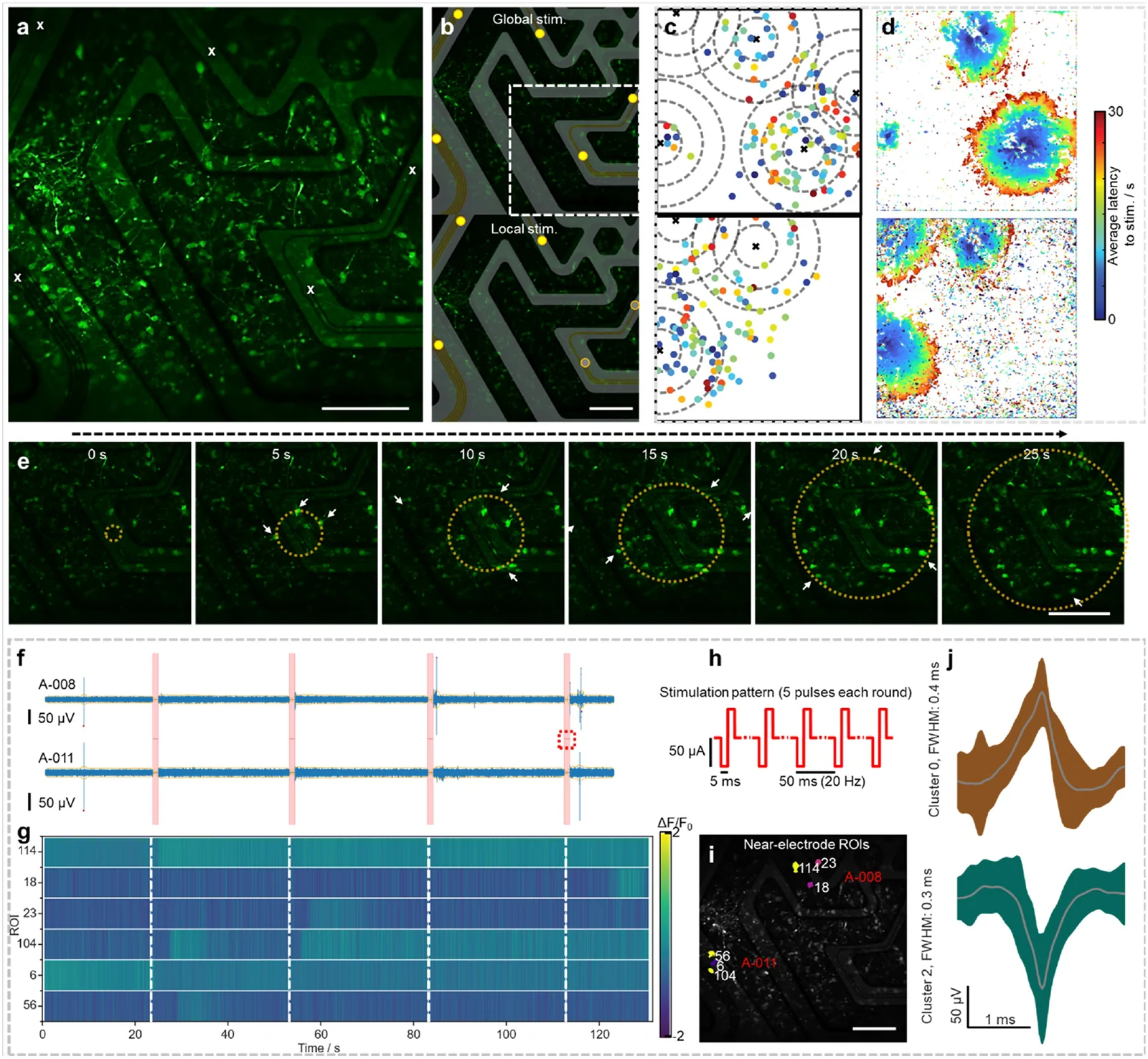
Simultaneous electrical stimulation and Ca^2+^ imaging using μEFPocket. **a.** An optical image showing Ca^2+^ fluorescence after electrical stimulation. Positions of electrodes are labeled as “x”. **b.-d.** The comparison between global electrical stimulation (top) and local stimulation (bottom). **b.** 2D schematic illustrations showing stimulating electrodes (in yellow). **c.** Average latency plot of responding neurons. **d.** Average latency plot of fluorescence imaging. **e.** Images showing network behavior after stimulation, corresponding to the labeled region in **b**. Post-stimulation time stamps are provided. Red dashed circles highlight the GCaMP8s activity propagation after electrical stimulation. **f.-g.** Synchronized electrical (**f**) and optical recording (**g**). The stimulation timestamps are labeled in **f-g** by red and white lines, respectively, with the simulation pattern illustrated in **h**. The fluorescence activity of several near-electrode ROIs (**i**) is analyzed and demonstrated in the spectrogram, showing clear responding activities after each round of stimulation. **j.** Selected spike waveforms recorded during stimulation. Scale bars: 200 μm.
